# The expression of immune response genes in patients with chronic Chagas disease is shifted toward the levels observed in healthy subjects as a result of treatment with Benznidazole

**DOI:** 10.3389/fcimb.2024.1439714

**Published:** 2024-07-23

**Authors:** Inmaculada Gómez, Adriana Egui, Génesis Palacios, Bartolomé Carrilero, Celia Benítez, Marina Simón, Manuel Segovia, Emma Carmelo, Manuel Carlos López, M. Carmen Thomas

**Affiliations:** ^1^ Departamento de Biología Molecular, Instituto de Parasitología y Biomedicina López-Neyra, Consejo Superior de Investigaciones Científicas, Granada, Spain; ^2^ Instituto Universitario de Enfermedades Tropicales y Salud Pública de Canarias, Universidad de La Laguna, La Laguna, Spain; ^3^ Unidad Regional de Medicina Tropical, Hospital Universitario Virgen de la Arrixaca, Murcia, Spain; ^4^ Departamento de Obstetricia y Ginecología, Pediatría, Medicina Preventiva y Salud Pública, Toxicología, Medicina Legal y Forense y Parasitología, Universidad de La Laguna, La Laguna, Spain

**Keywords:** chronic cardiac Chagas disease, *Trypanosoma cruzi*, transcriptional profile, highthroughput RT-qPCR, differential gene expression, immune response, immunological pathways, biomarkers

## Abstract

**Introduction:**

Chagas disease, caused by the *Trypanosoma cruzi* parasite infection, is a potentially life-threatening neglected tropical disease with a worldwide distribution. During the chronic phase of the disease, there exists a fragile balance between the host immune response and parasite replication that keeps patients in a clinically-silent asymptomatic stage for years or even decades. However, in 40% of patients, the disease progresses to clinical manifestations mainly affecting and compromising the cardiac system. Treatment is recommended in the chronic phase, although there are no early markers of its effectiveness. The aim of this study is to identify differential expression changes in genes involved in the immune response in antigen-restimulated PBMC from chronic patients with Chagas disease due to benznidazole treatment.

**Methods:**

Thus, high-throughput real-time qPCR analysis has been performed to simultaneously determine global changes in the expression of 106 genes involved in the immune response in asymptomatic (IND) and early cardiac manifestations (CCC I) Chagas disease patients pre- and post-treatment with benznidazole.

**Results and discussion:**

The results revealed that 7 out of the 106 analyzed genes were differentially expressed (4 up- and 3 downregulated) after treatment in IND patients and 15 out of 106 (3 up- and 12 downregulated) after treatment of early cardiac Chagas disease patients. Particularly in CCC I patients, regulation of the expression level of some of these genes towards a level similar to that of healthy subjects suggests a beneficial effect of treatment and supports recommendation of benznidazole administration to early cardiac Chagas disease patients. The data obtained also demonstrated that both in asymptomatic patients and in early cardiac chronic patients, after treatment with benznidazole there is a negative regulation of the proinflammatory and cytotoxic responses triggered as a consequence of *T. cruzi* infection and the persistence of the parasite. This downregulation of the immune response likely prevents marked tissue damage and healing in early cardiac patients, suggesting its positive effect in controlling the pathology.

## 1 Introduction

Chagas disease, also known as American trypanosomiasis, is caused by the protozoan parasite *Trypanosoma cruzi.* It is a potentially life-threatening neglected tropical disease that affects around 6–7 million people worldwide and causes between 10,000 and 14,000 deaths per year (Rassi et al., 2012; Organization Pan American Health and World Health Organization, 2021). The disease is endemic in Latin America, although migration from endemic areas to developed countries has caused the disease to spread to other regions such as the USA (Manne-Goehler et al., 2016), Canada, Australia, as well as many European countries, and some countries in Africa, the Eastern Mediterranean, and the Western Pacific (Schmunis, 2007; Schmunis and Yadon, 2010; Antinori et al., 2017).

After infection, the disease presents with an acute form characterized by high parasitemia and intense inflammatory infiltrates. Individuals who survive the acute phase of the infection develop a parasite-specific immune response that efficiently reduces parasite levels in the tissues and blood, leading to the chronic phase of the disease (Pérez-Molina and Molina, 2018). Chronic patients may remain asymptomatic for years, a phase known as the indeterminate (IND) form of the disease. However, approximately 30%–40% of them subsequently develop cardiac and/or gastrointestinal complications (Rassi et al., 2010). The most severe cases of cardiac alterations lead to the development of chronic chagasic cardiomyopathy (CCC), with which high mortality rates are associated (Morris et al., 1990; Rocha et al., 2003). Recent studies have shown that both patients in indeterminate and symptomatic early cardiac phases of Chagas disease (CCC I) show a differential gene expression profile in which several relevant immunological pathways seem to be activated or repressed (Gómez et al., 2021, 2023).

Since the beginning of the 1970s, there have been two drugs available for treating Chagas disease: benznidazole and nifurtimox. Both drugs are effective in acute and pediatric infections (Altcheh et al., 2005; Alonso-Vega et al., 2013; Viotti et al., 2014). However, apart from causing adverse events (up to 70% of the patients), which frequently force them to interrupt the treatment course, their efficacy in adult chronic patients is lower, variable, and difficult to evaluate due to the lack of a marker to define cure. The Pan-American Health Organization (PAHO) guidelines for the diagnosis and treatment of Chagas disease state that only seroconversion by conventional serology can be interpreted as an indicator of a parasitological cure (Pan American Health Organization, 2018). The limitation of seroconversion is that it takes 10–20 years to occur (Viotti et al., 2006; Fabbro et al., 2007). The detection of parasite DNA in the patient’s bloodstream by qPCR is consistent with treatment failure. By contrast, a negative PCR result may or may not be consistent with a cure due to the low parasitemia during the chronic phase (de Lima et al., 2023).

This situation highlights the need to elucidate the molecular mechanisms and signaling pathways that may be activated and regulated by treatment, with the aim of finding out and developing early biomarkers of treatment response that will facilitate patient follow-up. Different studies have reported several marker candidates, which together may fulfill acceptable criteria to indicate the efficacy of a trypanocidal treatment (Pinazo et al., 2014). Some of the antigens have exhibited promising results (Egui et al., 2019; Alonso-Padilla et al., 2021), indicating the need for further and larger studies to determine their potential role as biomarkers of treatment response. Some of these studies are currently in progress (Alonso-Vega et al., 2021).

The purpose of this study is to identify expression changes in genes involved in the immune response in PBMC restimulated with parasite’s soluble antigens due to benznidazole treatment in chronic patients with Chagas disease, differentiating those who are asymptomatic from those who present cardiac manifestations. Thus, genes coding for cytokines, chemokines, and their receptors, coestimulatory molecules, adhesion molecules, phenotype markers, transcription factors, cytotoxic molecules, inhibitory receptors and their ligands, molecules involved in apoptosis and senescence, and dendritic cell markers, among other molecules with immunological involvement, have been analyzed. To this end, a high-throughput qPCR platform was employed to simultaneously determine the expression level of 106 immune system-related genes in peripheral blood mononuclear cell (PBMC) samples from treated asymptomatic patients (posttreatment [IND-Pos]) and compared them with those who had not received treatment (IND patients pretreatment [IND-Pre]). Similarly, this response to treatment was also analyzed in Chagas disease patients at a more advanced stage of the disease, specifically with cardiac manifestations (CCC I, normal chest X-ray but abnormalities in the electrocardiogram, Kushnnir 1) (Kuschnir et al., 1985) before and after benznidazole treatment, CCC-Pre and CCC-Pos, respectively.

The results have shown that after benznidazole treatment, the expression pattern of genes involved in the cellular immune response is modulated in antigen-stimulated PBMC from patients with chronic Chagas disease, both asymptomatic and those with cardiac manifestations. Interestingly, the expression pattern of some of these genes reaches an expression pattern similar to that detected in healthy subjects. The study of the differential expression of these genes and the immune routes in which they are involved improves the comprehension of the effects of treatment at the immunological level. These genes could represent new potential biomarkers of therapeutic efficacy in chronic Chagas disease patients.

## 2 Materials and methods

### 2.1 Ethical considerations

The protocols employed in this study were approved by the Ethics Committees of the Consejo Superior de Investigaciones Científicas (Spain - Reference: 013/2020) and of the Hospital Virgen de la Arrixaca (Murcia, Spain - Reference: 2020-1-11-HCUVA). An informed consent was signed by all voluntary patients prior to their inclusion in the study.

### 2.2 Study cohort

Adult patients with chronic Chagas disease originating from endemic areas and residing in Spain included in this study were recruited, diagnosed, and clinically evaluated at the Hospital Virgen de la Arrixaca (Murcia, Spain). Patients were diagnosed according to the WHO criteria based on serological tests (ELISA Chagas, Ortho Clinical Diagnosis, and Inmunofluor Chagas, Biocientífica, Argentina) and characterized, according to the Kuschnir classification, as cardiac (CCC, G1 named CCC I) to those who had cardiac manifestations or IND (G0) due to the absence of cardiac or digestive manifestations (Gómez et al., 2021, 2023). Similarly, 24 CCC I and 61 IND patients who had received antiparasitic treatment (benznidazole; 5 mg/kg body weight per day for 60 days) were included. Blood samples from treated patients were collected from 6 to 12 months after treatment. Data concerning the age, gender, and country of origin of each of the subjects included in this study are detailed in **Table 1**.

**Table 1:** Epidemiological and demographic data of the study cohort.

Patients group		Origin (%)	Age (years)		Sex [%Female(F) /Male (M)]
			Mean (+ SD)	Range	
**Healthy donors (n=34)**	**From non- endemic area (n=20)**	Spain (100%)	37.6 (12.8) 37.5 (23)	22-56	60% F
						40% M
					**From endemic area (n=14)**		Colombia (28.6%)	37.5 (8.4) 38 (8)	21-54	64.3% F
						Venezuela (14.3%)				
	Chile (7.1%)									
	Panama (7.1%)	35.7% M							
	Ecuador (7.1%)								
	ND (35.7%)								
	**Post-treatment IND patients (n=61)**		Bolivia (96.7%)	37.1 (8.6)	21-57	70.5% F 29.5% M
			El Salvador (1.6%)			
Paraguay (1.6%)						
**Post-treatment CCC patients(n=24)**		Bolivia (83.3%)	32.8 (9)	19-55	62.5% F 37.5% M
		Paraguay (4.2%)			
		ND (12.5%)			

ND, no data.

### 2.3 Isolation of peripheral blood mononuclear cells

PBMCs were isolated from 20- to 30-mL blood samples collected aseptically from each subject into EDTA-coated tubes. These PBMCs were purified and cryopreserved following the previously described protocol (Marañón et al., 2011).

### 2.4 Isolation of *Trypanosoma cruzi*-soluble antigens

Soluble antigens from *T. cruzi* (*Tc*SA) SOL strain (MHOM/ES/2008/SOL; DTU V) isolated from an infant (referred to as infact 1) infected by congenital transmission in Spain, were extracted following the protocol previously described (Egui et al., 2017; Gómez et al., 2021) and employed for *in vitro* stimulation of patients PBMCs. The protein concentration of the extracts was determined using a microbicinchoninic acid (BCA) protein assay kit (Thermo Fisher Scientific Inc., Waltham, MA, USA), with the protein profile analyzed by SDS-PAGE and subsequent Coomassie blue staining.

### 2.5 Thawing and stimulation of peripheral blood mononuclear cells

Cryopreserved PBMCs were thawed and stimulated following a previously described laboratory protocol (Egui et al., 2018; Pérez-Antón et al., 2018; Gómez et al., 2021). Briefly, PBMCs were rapidly thawed in a 37°C water bath, transferred to a tube containing 10 mL of RPMI-1640 (supplemented with 2 mM l-glutamine, 10% iFBS, and 50 µg/mL of gentamicin), centrifuged at 453 rcf for 10 min, and suspended in 2 mL of supplemented RPMI-1640 medium. Cell viability was analyzed in each sample by cell staining with trypan blue, exhibiting a mean viability of 80%. Viable PBMCs were seeded at a concentration of 7.5–8.5 × 10^6^ cells/mL in 12-well plates in a maximum volume of 3.5 mL/well, cultured for 4 h at 37°C in 5% CO_2_, and subsequently stimulated with *Tc*SA (10 μg/mL) and cultured for 14–14.5 h at 37°C in 5% CO_2_.

### 2.6 RNA isolation, quantification, and quality analysis

Total RNA isolation from stimulated PBMCs was performed using the RNeasy Plus Mini kit (QIAGEN Inc., Valencia, CA, USA), eliminating the genomic DNA and obtaining mRNA-enriched samples, according to the manufacturer’s indications. Due to the quantity of RNA required to carry out cDNA synthesis for high-throughput RT-qPCR and the limited number of cells isolated from the small volume of blood samples of particular patients, in some cases, it was necessary to blend cells from some patients. In the cohort of CCC I patients, 18 samples corresponded to individual patients and three to a mixture of two patients. In the case of IND patients, 35 samples corresponded to individual subjects, seven to a mixture of two patients, and four to a mixture of three patients. The NanoDrop 1000 spectrophotometer (Thermo Fisher Scientific Inc., Waltham, MA, USA) and Qubit fluorometer (Invitrogen, Carlsbad, CA, USA) were employed to quantify and determine RNA purity. The quality of the extracted mRNA was determined by analyzing its integrity with the Bioanalyzer 2100 (Agilent Technologies, Santa Clara, USA) using the RNA 6000 Nano kit (Agilent Technologies, Santa Clara, USA). All RNA samples included in this study had high quality and integrity, with an RNA integrity number (RIN) ranging from 7.4 to 10.

### 2.7 Reverse transcription and high-throughput real-time quantitative PCR

A High-Capacity cDNA Reverse Transcription Kit (Applied Biosystems, Foster City, CA, USA) was used to perform the reverse transcription as previously described (Gómez et al., 2021). Briefly, real-time qPCR was performed using OpenArray^®^ plates with TaqMan probes (Thermo Fisher Scientific) for the amplification of 106 specific immune-related genes and six endogenous reference genes. All primers and probes were commercially designed by Thermo Fisher Scientific (Gómez et al., 2023) and are listed in **Supplementary Table S1**. The PCR mixture was prepared according to the manufacturer’s instructions. Each amplification reaction was performed in triplicate. The thermal cycle (95°C for 15 s, 60°C for 1 min, for 40 cycles) and fluorescence detection were performed with the QuantStudio™ 12K Flex Real-Time PCR System (Thermo Fisher Scientific) according to the manufacturer’s protocol. The quantitative cycle (Cq) values produced by this platform are already corrected for the efficiency of the amplification (Hernandez-Santana et al., 2016).

### 2.8 Data processing

The arithmetic average Cq values for each qPCR run were employed for data analysis after they had been exported from QuantStudio™ 12K Flex Real-Time PCR System. To identify the genes with the highest expression stability, GeNorm (Vandesompele et al., 2002), NormFinder (Andersen et al., 2004), and RefFinder (Xie et al., 2012) (heartcure.com.au), which integrate the four algorithms GeNorm (Vandesompele et al., 2002), NormFinder (Andersen et al., 2004), BestKeeper (Pfaffl et al., 2004), and ΔCt method (Silver et al., 2006), were applied to the 112 analyzed genes. *STAT3*, *IL10RA*, and *IFNAR* were the three genes that showed a more stable expression, all with geNorm *M*-values < 0.5 (which is the standard cutoff for reference gene selection). These genes were selected as reference genes and used for data set normalization employing GenEx software (v.6, MultiD), obtaining normalized relative quantity (NRQ) values. The six constitutive and candidate reference genes included in the panel (*ACTB*, *B2M*, *GAPDH*, *HPRT1*, *PGK1*, and *TBP*) were disregarded as they had worse stability values than *STAT3*, *IL10RA*, and *IFNAR* in all the algorithms employed. For comparative analyses, the Cq values for each qPCR and the correspondent NRQ values obtained from healthy donors (HD) were taken into consideration (Gómez et al., 2021).

### 2.9 Enrichment analysis

Gene set enrichment analysis was carried out on the NRQ values of each group of patients as a whole, employing GSEA 4.1.0 software (Mootha et al., 2003; Subramanian et al., 2005). For the enrichment analysis, canonical pathways gene sets from the BioCarta pathway database included in *C2: curated gene sets collection in Molecular Signatures Database* (*MSigDB*) (Subramanian et al., 2005; Liberzon et al., 2011) were selected. GSEA parameters: permutations = 100,000, permutation type: phenotype (sample *n* > 7), enrichment statistic: weighted, metric for ranking genes: *t*-test, max size: 500, min size: 2.

### 2.10 Statistical analyses

All statistical analyses were performed using SPSS v25 software (IBM Corp., Armonk, NY, USA) and GraphPad Prism v8 (GraphPad Software, San Diego, CA, USA). Kolmogorov–Smirnov and Shapiro–Wilk tests (α = 0.05) were employed to check data normality, and a two-tailed unpaired *t*-test or a two-tailed Mann–Whitney *U* test, depending on whether data had or not a normal distribution, respectively, was used to determine the statistical significance, considering *p* < 0.05 as statistically significant. Differentially expressed genes between chronic IND-treated and untreated patients, as well as between early cardiac treated and untreated patients, were identified using two parameters: the fold-change of gene expression (FC) and the statistical significance (*p*-value). FC was calculated as the ratio between the average gene expression of the groups of patients (IND-Pos/IND-Pre, CCC-Pos/CCC-Pre), indicating how many times a specific gene is expressed in one group *versus* another. To be able to determine the FC when the average gene expression level was 0 in one of the compared groups, the number 0 was replaced by 0.000001 (this was applied for *IL17A* in the CCC-Pos group). To visualize changes, volcano plots were made from the –log_10_ *p-*value (determined by an unpaired *t*-test) plotted on the *y*-axis and log_2_ of FC on the *x*-axis. Genes passing both biological significance (log_2_ of FC > 0.6 or ≤ 0.6, equivalent to FC > 1.5 or < 0.66) and statistical significance (–log_10_ *p* > 1.3, equivalent to *p* = 0.05) thresholds were shown in red and blue, attending to their up- and downregulation, respectively. STRING v11 (Szklarczyk et al., 2019) was employed to generate interaction networks between differentially expressed genes. Active interaction sources, including experiments, databases, cooccurrence, gene fusion, and neighborhood, were applied to construct protein–protein interaction networks, limiting species to *Homo sapiens* and interaction scores > 0.7. Principal component analysis (PCA) was performed for multivariate analysis on NRQ values to determine the structure of the dataset. Differences in scores of plotted principal components between the groups were confirmed by an unpaired *t*-test or a Mann–Whitney *U* test, as appropriate, employing SPSS v25 software.

## 3 Results

To analyze the immune response of patients with Chagas disease after benznidazole treatment and its relationship with disease control or progression, comparative analyses focusing on the global changes in the expression of 106 genes involved in the immune response have been performed in patients with Chagas disease pre- and posttreatment with benznidazole. The study has been carried out in patients in the IND asymptomatic phase and patients with cardiac symptomatology (Kuschnir I) (CCC I), given that the efficacy of the treatment may vary according to the phase of the disease at the time of drug administration. The comparative analyses were carried out in all cases, considering the NRQ values of PBMC cells from these patients stimulated with *T. cruzi* antigens and also those from healthy subjects (Gómez et al., 2021). Despite the comparative analyses performed among groups of patients being carried out as a whole and not as individual subject samples (untreated versus treated IND and untreated versus treated CCC I), the potential influence on the gene expression pattern of samples obtained from individual samples or from blended samples was analyzed. Thus, PCA were performed considering samples from individual IND-treated patients as a different group of the blended samples of treated IND patients and also individual treated CCC I patients as a different group of the treated CCC I patients blended samples. The obtained results showed that samples from the IND and CCC-treated patients conserved the same distribution independently, whether they came from individual samples or had been blended (**Supplementary Figure S1**). The same level of gene expression in both the individual and blended samples from IND and CCC I-treated samples was supported by a two-tailed unpaired *t*-test, which showed that there were no statistically significant differences between the scores obtained in the two groups for each principal component.

### 3.1 Analysis of differentially expressed genes in chronic patients in the indeterminate phase before and after treatment

**Figure 1:**
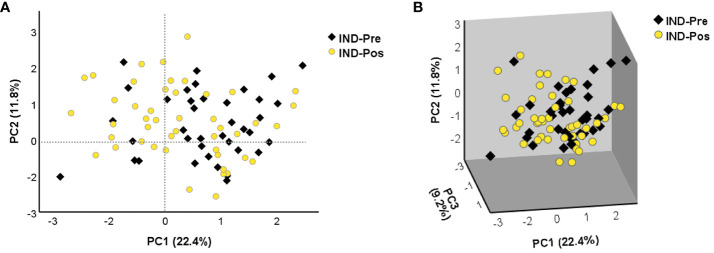
The principal component analysis applied to the NRQ values of 106 genes from IND-Pre and IND-Pos patients. **(A)** PCA score plot of principal components 1 (PC1) and 2 (PC2) on the *x*-axis and *y*-axis, respectively. **(B)** 3D scatter plot showing the three principal components 1 (PC1), 2 (PC2), and 3 (PC3). The proportion of variance explained by each principal component is indicated as a percentage on the axis next to the corresponding principal component.

**Figure 2:**
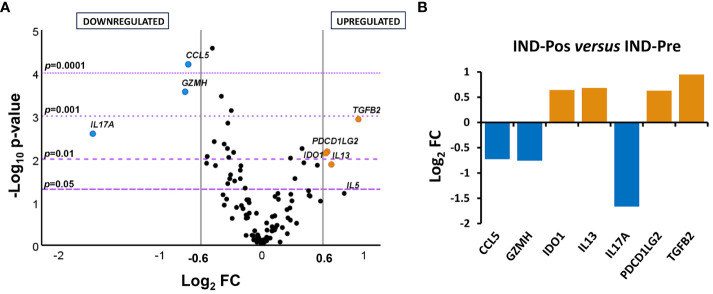
Differential gene expression levels in posttreatment versus pretreatment indeterminate Chagas disease patients. **(A)** Volcano plot showing the differential expression level of the 106 analyzed genes in indeterminate Chagas disease patients pretreatment (IND-Pre, *n* = 39) and posttreatment (IND-Pos, *n* = 46). The *x*-axis represents log_2_ of expression fold change between IND-Pos and IND-Pre (log_2_FC). The *y*-axis corresponds to the statistical significance, expressed as the negative logarithm of the *p*-value (–log_10_ *p*-value). The purple horizontal lines indicate the cutoffs for the statistical significance (corresponding to *p* = 0.05, *p* = 0.01, *p* = 0.001, and *p* = 0.0001). The gray vertical lines represent the log_2_FC of − 0.6 and 0.6 (corresponding to FC of 0.66 and 1.5, respectively) used as biological thresholds to identify differentially expressed genes. Negative values correspond to downregulated genes (blue dots) and positive values to upregulated genes (orange dots) in IND-Pos compared to IND-Pre. Black dots represent nondifferentially expressed genes. **(B)** Expression level of seven differentially expressed genes with statistical significance in IND-Pos versus IND-Pre patients. Each plot bar corresponds to the gene referred to at the bottom of the graph (*x*-axis). The *y*-axis represents the log_2_ of expression fold change (log_2_FC) for each gene. Positive values (orange bars) indicate upregulated genes, and negative values (blue bars) indicate downregulated genes in IND-Pos compared to IND-Pre subjects.

To determine the distribution of the gene expression data set [NRQ values of IND-Pre (*n* = 39) versus IND-Pos (*n* = 46)], a principal component analysis was performed as a first approximation. As shown in **Figure 1**, principal component 1 (PC1) and principal component 2 (PC2) explained the highest percentages of total variance with values of 22.4% and 11.8%, respectively (**Figure 1A**). Also, a 3D representation for PCA including principal component 3 (PC3) showed PC3 explains 9.2% of the total variance (**Figure 1B**). The results showed that the scores obtained for PC1 in the pretreatment IND patients were mostly positive, whereas in the posttreatment patients, they were distributed along the entire *x*-axis where this component was represented. The observed differences in PC1 scores between the two groups of subjects were confirmed by a two-tailed unpaired *t*-test, which indicated that there was a statistically significant differential expression profile before and after treatment in IND patients (*p* = 0.025). Additionally, this same statistical test was applied to corroborate that principal components 2 and 3 were not responsible for the differences observed between the two groups (PC2 *p* = 0.945, PC3 *p* = 0.354). The 24 genes correlated with PC1 with high factor loadings are listed in **Supplementary Table S2**. Subsequently, to determine the influence of treatment, the gene expression level of the 106 genes under study in IND patients untreated and treated with benznidazole was comparatively analyzed. The results revealed that seven out of the 106 analyzed genes were differentially expressed between treated and untreated IND patients with statistical significance (*p* < 0.05), as observed in the volcano plot constructed from the FC and statistical significance for each gene (**Figure 2A**). Specifically, four genes (*IDO1*, *IL13*, *PDCD1LG2*, and *TGFB2*) were upregulated in IND-Pos patients versus IND-Pre patients (FC > 1.5; orange dots). Conversely, three out of the seven genes (*CCL5*, *GZMH*, and *IL17A*) were downregulated in the IND-Pos patients compared with IND-Pre (FC < 0.66; blue dots).

The differences detected in the gene expression level of the genes whose expression was modified by the treatment were quantified by representing the log_2_ of fold change values obtained for each gene in a bar graph (**Figure 2B**). The results indicated that up- and downregulated genes (log_2_ FC > 0.6 (orange bars) or ≤ 0.6 (blue bars), respectively) had fairly similar log_2_ FC values according to the same level of expression change, with the exception of the *IL17A* gene. Thus, *IL17A* showed a log_2_ FC value below − 1.5, which is equivalent to at least a threefold lower expression level in the post-treatment IND patients compared to the pre-treatment IND patients.

### 3.2 Identification of differentially expressed genes in chronic cardiac patients (CCC I) before and after treatment

**Figure 3:**
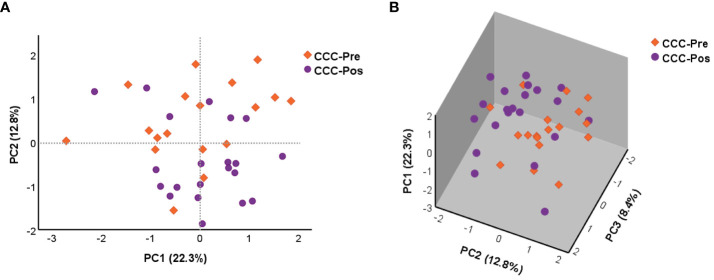
Principal component analysis (PCA) was applied to the NRQ values of 106 genes from CCC-Pre and CCC-Pos. **(A)** PCA score plot of principal components 1 (PC1) and 2 (PC2) on the *x*-axis and *y*-axis, respectively. **(B)** 3D scatter plot showing the three principal components 1 (PC1), 2 (PC2), and 3 (PC3). The proportion of variance explained by each principal component is indicated as a percentage on the axis next to the corresponding principal component.

The effect of benznidazole treatment on the global changes in the expression level of the 106 genes under study was extended to chronic Chagas disease patients at the early stage of the cardiac phase (CCC I). A principal component analysis was again performed using the NRQ values of patients with untreated CCC Chagas disease (CCC-Pre, *n* = 18) as well as the group of CCC patients who had received treatment (CCC-Pos, *n* = 21) (**Figure 3**). PCA results indicated, as shown in **Figure 3A**, that PC1 and PC2 explained 22.3% and 12.8% of the total variance, respectively. PC3, which explained 8.4% of the variance, was also plotted together with PC1 and PC2 in a 3D scatter plot (**Figure 3B**). This analysis indicated that most samples of each group tended to cluster together along the axis where PC2 was represented. These observed differences in acquired scores for PC2 between pre- and posttreatment CCC patients were confirmed by a two-tailed unpaired *t*-test, which indicated that there was a statistically significant differential expression profile between the two groups of patients for 13 genes correlated with PC2 (*p* = 0.003). A two-tailed unpaired *t*-test was also applied to the scores acquired for PC1 and PC3, which showed that they did not participate in the observed differences (PC1 *p* = 0.641; PC3 *p* = 0.914). The 13 genes out of the 106 analyzed correlated with high factorial loading with PC2 are listed in **Table 2**.

**Table 2:** PC2-correlated genes.

Gene	Factor loading for PC2
*FASLG*	0.879
*CCL5*	0.787
*B3GAT1*	0.747
*HAVCR2*	0.741
*PRF1*	0.729
*GZMH*	0.708
*GZMA*	0.680
*TGFBR1*	0.642
*IL10RA*	0.633
*NCAM1*	0.625
*GZMB*	0.625
*LAG3*	0.623
*STAT1*	− 0.680

Genes with factor loading of principal component 1 (PC1) higher than 0.6 or lower than − 0.6 from the principal component analysis (PCA) applied to the normalized relative quantities (NRQ) of cardiac Chagas disease patients pre- and posttreatment.

**Figure 4:**
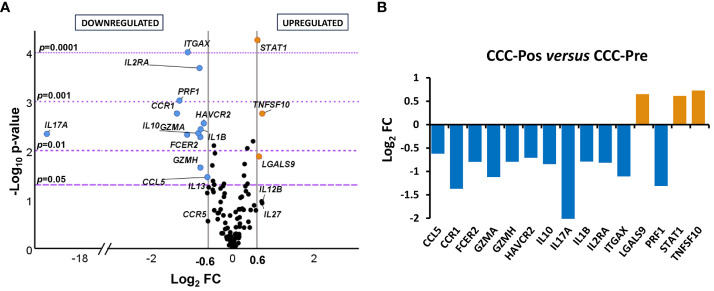
Differential gene expression levels in posttreatment versus pretreatment cardiac Chagas disease patients. **(A)** Volcano plot showing the differential expression level of the 106 analyzed genes in cardiac Chagas disease patients pretreatment (CCC-Pre, *n* = 18) and posttreatment (CCC-Pos, *n* = 21). The *x*-axis represents log_2_ of expression fold change between CCC-Pos and CCC-Pre (log_2_FC). The *y*-axis corresponds to the statistical significance, expressed as the negative logarithm of the *p*-value (–log_10_ *p*-value). The purple horizontal lines indicate the cutoffs for the statistical significance (corresponding to *p* = 0.05, *p* = 0.01, *p* = 0.001, and *p* = 0.0001). The gray vertical lines represent the log_2_FC of − 0.6 and 0.6 (corresponding to FC of 0.66 and 1.5, respectively) used as biological thresholds to identify differentially expressed genes. Negative values correspond to downregulated genes (blue dots) and positive values to upregulated genes (orange dots) in CCC-Pos compared to CCC-Pre. Black dots represent nondifferentially expressed genes. **(B)** Expression level of 15 differentially expressed genes with statistical significance in CCC-Pos versus CCC-Pre patients. Each plot bar corresponds to the gene referred to at the bottom of the graph (*x*-axis). The *y*-axis represents the log_2_ of expression fold change (log_2_FC) for each gene. Positive values (orange bars) indicate upregulated genes, and negative values (blue bars) indicate downregulated genes in CCC-Pos compared to CCC-Pre.

Analysis of the genes differentially expressed in the pre- and posttreatment CCC patients indicated that 15 out of the 106 genes under study were differentially expressed between the two groups of patients with statistical significance (**Figure 4A**). Thus, 12 out of these 15 differentially expressed genes (*CCL5*, *CCR1*, *FCER2*, *GZMA*, *GZMH*, *HAVCR2*, *IL10*, *IL17A*, *IL1B*, *IL2RA*, *ITGAX*, and *PRF1*) were downregulated (log_2_ FC ≤ 0.6) and three genes (*LGALS9*, *STAT1*, and *TNFSF10*) upregulated (log_2_ FC > 0.6) in CCC-Pos compared to CCC-Pre, as seen in the volcano graph plotting the log_2_ FC of each gene versus statistical significance.

The significance of the differences observed in the expression level of each gene reached a –log_10_ *p*-value greater than 1.3, equivalent to a *p* < 0.05. To easily visualize the intensity of the changes, the log_2_ FC values of the biologically and statistically differentially expressed genes (log_2_ FC > 0.6 or ≤ 0.6 and *p*-value < 0.05) in posttreatment CCC patients versus pretreatment CCC patients were represented in a bar graph. As shown in **Figure 4B**, five genes were particularly downregulated in CCC-Pos patients (*CCR1*, *GZMA*, *IL17A*, *ITGAX*, and *PRF1*), exhibiting a log_2_ FC value lower than – 1, which resulted in a reduction of at least half the amount of expression *versus* CCC-Pre patients. In the case of *IL17A*, no expression was detected in any of the samples from the CCC-Pos patient group.

**Figure 5:**
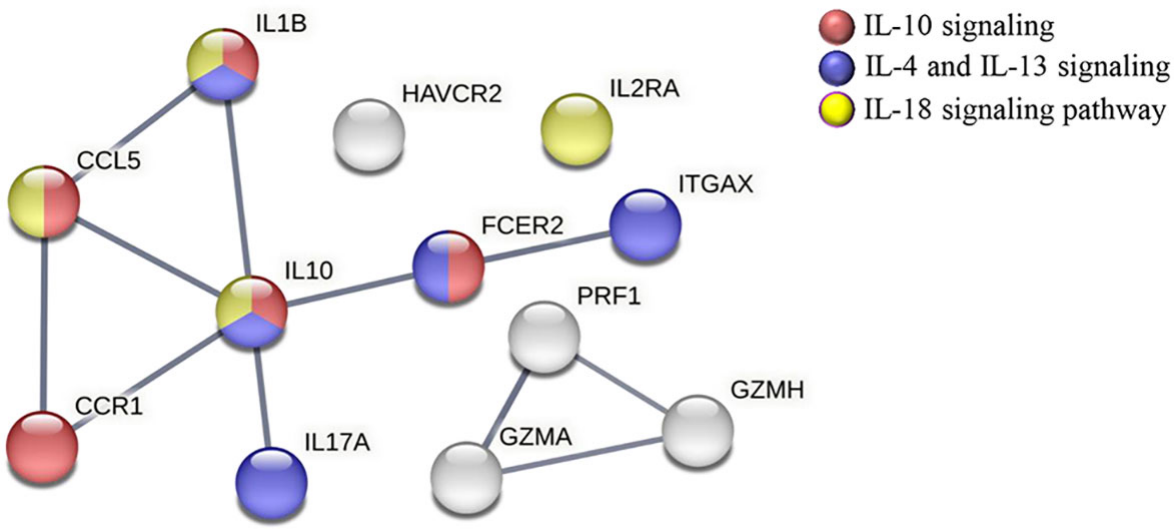
STRING protein–protein interaction analysis representing the proteins encoded by downregulated genes in CCC-Pos versus CCC-Pre. The linkage of each node represents the interactions (edges) reported for these molecules in different immunological processes/pathways; PPI enrichment *p*-value < 1.0*e*−16. Network edges represent confidence (line thickness indicates the strength of data support). PPI network was constructed, setting the confidence score threshold at the high level (0.7) and active interaction sources, including data from published experiments, databases, cooccurrence, gene fusion, neighborhood and coexpression, and species limited to *Homo sapiens*. Proteins are described to be involved in specific pathways: IL-10 signaling (HSA-6783783; FDR = 5.00*e*−08) (red), IL-4 and IL-13 signaling (HSA-6785807; FDR = 2.33*e*−06) (blue), and IL-18 signaling pathway (WP4754; FDR = 0.0029) (yellow).

To predict the specific biological function in which the 12 downregulated genes in the CCC-Pos versus CCC-Pre patients may be involved, a protein–protein interaction (PPI) network was constructed using the STRING platform, only considering a high level of confidence for the predicted interactions. The results revealed that 12 proteins encoded by the set of differentially downregulated genes in CCC-Pos versus CCC-Pre had a high degree of interaction, reaching up to 11 interactions (edges) with a PPI enrichment *p*-value < 1.0*e*−16. In addition, the analysis showed that no interaction would be expected for a random set of proteins of the same size by chance. The role of these proteins in relevant immunological pathways was also analyzed in the obtained STRING network. As observed in **Figure 5**, the results showed that some proteins participated in more than one pathway. Thus, five proteins (CCL5, CCR1, FCER2, IL10, and IL1B) were predicted to participate in the “IL-10 signaling” pathway (HSA-6783783; FDR = 5.00*e*−08; red nodes), five proteins (FCER2, IL10, IL17A, IL1B, and ITGAX) in the “IL-4 and IL-13 signaling” pathway (HSA-6785807; FDR = 2.33e-06; blue nodes), and four proteins (CCL5, IL10, IL1B, and IL2RA) in the “IL-18 signaling pathway” (WP4754; FDR = 0.0029; yellow nodes).

**Figure 6:**
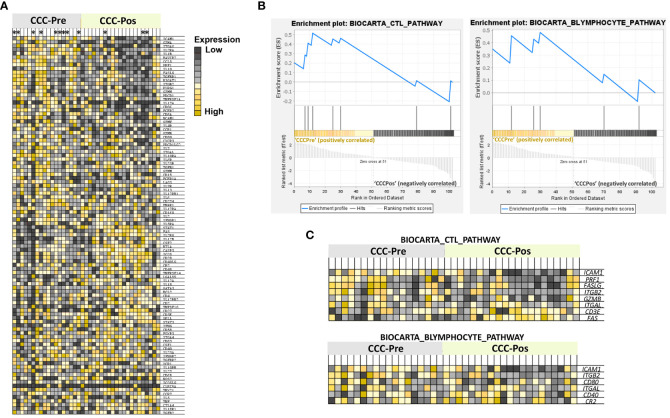
Gene set enrichment analysis (GSEA). **(A)** Heatmap of the top-100 genes determined by GSEA in cardiac Chagas disease patients pre- (CCC-Pre) and posttreatment (CCC-Pos). The values of the expression level of each gene are represented as colors, ranging from dark red to dark blue, based on the highest and lowest NRQ values of each gene, respectively. The genes represented in vertical order from top to bottom are as follows: *ICAM1*, *GZMA*, *ITGAX*, *IL2RA*, *IL1B*, *HAVCR2*, *CCL5*, *PRF1*, *IL10*, *FASLG*, *TGFBR1*, *B3GAT1*, *ITGB2*, *KLRG1*, *GZMH*, *PDCD1*, *TNFRSF1A*, *IL17A*, *CD86*, *FCER2*, *CD8A*, *NCAM1*, *GZMK*, *IL4R*, *CCR1*, *GZMB*, *CD80*, *CXCR3*, *PDCD1LG2*, *IL7*, *ITGAL*, *IL10RA*, *IL6R*, *IL23R*, *TGFB1*, *GZMM*, *CD19*, *FCER1A*, *LAG3*, *IL7R*, *IL13*, *IL12RB1*, *IL6*, *CD274*, *FNAR1*, *IL17RA*, *CD160*, *IL2*, *IFNGR1*, *IL5RA*, *STAT1*, *FAS*, *IL2RG*, *IL12B*, *CSF2*, *BTLA*, *CASP3*, *ICOS*, *CD28*, *CD40LG*, *CR2*, *CD48*, *TNFRSF14*, *LGALS9*, *IL12A*, *IL18*, *GATA3*, *BCL2*, *CD4*, *IL12RB2*, *CD2*, *TNFSF10*, *CD27*, *CD3E*, *SELL*, *STAT3*, *IFNG*, *CD58*, *FOXP3*, *ITGA4*, *CD83*, *CSF1*, *CD40*, *IL23A*, *IFNGR2*, *TGFBR2*, *XCR1*, *IL10RB*, *IL27*, *CD69*, *NOS2*, *ICOSLG*, *CLEC9A*, *TBX21*, *CCR7*, *IL5*, *TNF*, *CTLA4*, *IL18R1*, and *TGFB2*. Patients whose samples were merged have been marked with an asterisk at the top of the heat map. **(B)** Enrichment plots of gene sets from “CTL mediated immune response against target cells (BIOCARTA_CTL_PATHWAY)” and “B Lymphocyte Cell Surface Molecules (BIOCARTA_BLYMPHOCYTE_PATHWAY)” pathways in CCC-Pre and CCC-Pos. The green curve denotes the enrichment score (ES) curve. **(C)** GSEA heatmaps of gene sets from BIOCARTA_CTL_PATHWAY and BIOCARTA BLYMPHOCYTE PATHWAY. Parameters set for GSEA were: *Molecular Signatures Database* (MSigDB) BioCarta gene set collection, permutations = 100,000, permutation type: phenotype, enrichment statistic: weighted, metric for ranking genes: *t*-test, max size: 500, min size: 2.

To gain insight into the immunological pathways affected by benznidazole treatment in CCC I, a gene set enrichment analysis (GSEA) was applied to the NRQ values of untreated and benznidazole-treated CCC I. Therefore, a heatmap was obtained showing the top 100 genes that undergo the greatest expression changes between both groups of samples from CCC patients (**Figure 6A**). In general terms, this graph allowed us to observe that the expression of several genes under study was downregulated in the majority of the samples of CCC I patients after treatment. By contrast, the expression of other genes was higher in the samples of patients after treatment compared to those collected before treatment. In addition, to obtain enrichment plots able to illustrate a positive or negative correlation between the specific gene sets and the samples from CCC patients pre- and posttreatment, a Molecular Signatures Database (MSigDB) BioCarta gene set collection (Subramanian et al., 2005; Liberzon et al., 2011) was employed (**Figures 6B, C**). As shown in **Figure 6B**, GSEA revealed a positive correlation between CCC-Pre and the immune pathways “CTL mediated immune response against target cells (BIOCARTA_CTL_PATHWAY)” and “B lymphocyte cell surface molecules (BIOCARTA_BLYMPHOCYTE_PATHWAY)” with a normalized enrichment score (NES) of 1.17 and 1.02, respectively. The expression of the genes involved in these two enriched pathways is shown in GSEA heatmaps (**Figure 6C**). Thus, the *ICAM1*, *PRF1*, *FASLG*, *ITGB2*, *GZMB*, *ITGAL*, *CD3E*, and *FAS* genes are included in the BIOCARTA_CTL_PATHWAY pathway. The core enrichment of this pathway includes *ICAM1*, *PRF1*, *FASLG*, and *ITGB2*. Similarly, *ICAM1*, *ITGB2*, *CD80*, *ITGAL*, *CD40*, and *CR2* genes participate in the BIOCARTA_BLYMPHOCYTE_PATHWAY pathway, including the core enrichment of this pathway to *ICAM1*, *ITGB2*, *CD80*, and *ITGAL* genes.

### 3.3 Benznidazole treatment modulates gene expression in Chagas disease patients toward the expression level of healthy donors, depending on the severity of the disease

To identify the changes produced in the DEG related to the effect of the treatment and the progression and severity of the disease, the average expression of each DEG, shown as NRQ values, was compared between indeterminate and cardiac Chagas’ disease patients before and after treatment, taking also into consideration the healthy subjects (Gómez et al., 2021). As shown in **Table 3**, the expression of certain genes such as *CCL5*, *FCER2*, *GZMH*, *IDO1*, *IL10*, *IL13*, *IL1B*, *IL2RA*, *LGALS9*, *PDCD1LG2*, *PRF1*, *STAT1*, *TGFB2*, and *TNFSF10* was increased in IND-Pre and also in CCC-Pre patients with respect to HD (with the exception of the *LGALS9* gene). Most of these genes were even more overexpressed in CCC-Pre patients with respect to IND-Pre (*FCER2*, *GZMH*, *IDO1*, *IL10*, *IL13*, *IL1B*, *IL2RA*, *PDCD1LG2*, *PRF1*, and *TGFB2*). However, a decrease in the expression of the majority of these genes (with the exception of *IDO1*, *TGFB2*, and *LGALS9*, whose expression increased) was observed in CCC patients after treatment (CCC-Pos). This decrease reached an expression value closer to the expression values detected in healthy donors in the cases of *CCL5*, *FCER*2, *GZMH*, *IL10*, *IL13*, *IL1B*, *IL2RA*, *PDCD1LG*2, and *PFR1*. In contrast, the expression level of *LGALS9* in CCC-Pre was lower than that detected in HD and IND-Pre patients, although its expression increased in CCC-Pos patients, reaching an expression level closer to that detected in HD and IND-Pre patients.

**Table 3:** Expression level of differentially expressed genes in IND and/or CCC I Chagas disease patients pre- and posttreatment.

Gene	HD	IND-Pre	IND-Pos	CCC-Pre	CCC-Pos
***CCL5***	**1.188**	**1.485**	**0.898**	**1.298**	**0.844**
*CCR1*	3.339	1.166	1.102	1.156	0.447
***FCER2***	**0.684**	**1.214**	**1.323**	**1.754**	**1.012**
*GZMA*	2.139	1.262	0.865	1.524	0.701
***GZMH***	**0.828**	**1.577**	**0.934**	**1.641**	**0.947**
*HAVCR2*	2.351	0.939	0.970	1.149	0.703
*IDO1*	0.408	1.137	1.775	1.305	1.588
***IL10***	**0.843**	**1.050**	**1.207**	**1.605**	**0.894**
***IL13***	**0.694**	**0.992**	**1.593**	**1.399**	**0.908**
*IL17A*	0.604	0.503	0.159	0.462	0.000
***IL1B***	**0.671**	**1.249**	**1.517**	**1.903**	**1.103**
***IL2RA***	**0.634**	**1.109**	**1.350**	**1.439**	**0.819**
*ITGAX*	3.214	0.966	1.022	0.947	0.440
***LGALS9***	**0.924**	**1.004**	**1.465**	**0.706**	**1.109**
***PDCD1LG2***	**1.033**	**0.921**	**1.424**	**1.232**	**0.889**
*PRF1*	0.928	1.121	1.056	1.534	0.619
*STAT1*	0.499	1.353	1.451	1.144	1.749
*TGFB2*	0.425	0.625	1.208	0.751	1.118
*TNFSF10*	0.592	1.075	1.151	0.825	1.368

Average expression values expressed as normalized relative quantities (NRQ) identified in healthy donors (HD), indeterminate patients pre- (IND-Pre) and posttreatment (IND-Pos), and cardiac patients pre- (CCC-Pre) and posttreatment (CCC-Pos). In bold are the genes whose posttreatment expression has a value that tends to that of healthy donors and/or, in the case of CCC, to the values of IND patients.

**Figure 7:**
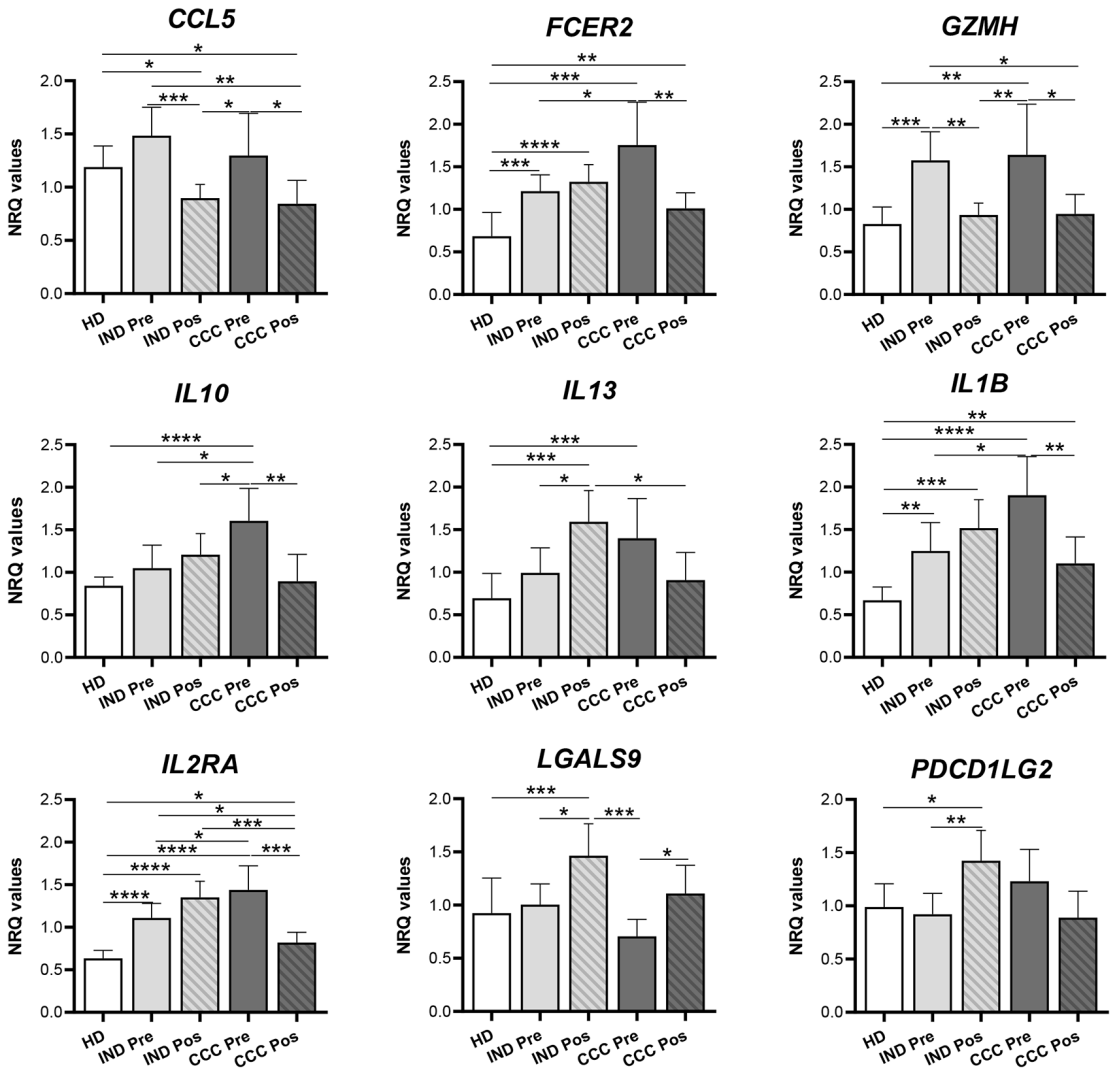
Differentially expressed genes after benznidazole treatment in indeterminate and/or cardiac Chagas disease patients. Expression level of *CCL5*, *FCER2*, *GZMH*, *IL10*, *IL13*, *IL1B*, *IL2RA*, *LGALS9*, and *PDCD1LG2* genes measured as mean normalized relative quantities (NRQ) in healthy donor (HD), indeterminate patients pre- (IND-Pre) and posttreatment (IND-Pos), and cardiac patients pre- (CCC-Pre) and posttreatment (CCC-Pos). Statistically significant differences, determined by a two-tailed Mann–Whitney *U* test or a two-tailed unpaired *t*-test as appropriate, are indicated (^*^ *p* < 0.05; ^**^ *p* < 0.01; ^***^ *p* < 0.001; ^****^ *p* < 0.0001).

The study was subsequently focused on the expression of the nine genes that in CCC patients showed a decrease (or an increase in the case of *LGALS9*) in their expression after treatment, reaching values similar to those in HD or IND. The result was represented in bar graphs, taking into consideration the expression level of these genes in healthy subjects and the statistical significance of the observed differences. As observed in **Figure 7**, *CCL5* and *GZMH* were upregulated in Chagas disease patients, both IND-Pre and CCC-Pre versus HD. Following benznidazole treatment, it was observed a significant reduction in the expression of those genes in both groups of patients (*p*-values = 0.001, 0.01, and 0.05). Another group of genes (*FCER2*, *IL10*, *IL13*, *IL1B*, and *IL2RA*) showed significant upregulation along disease progression, particularly elevated in the CCC stage compared to HD (with important statistical significance for all of them with *p*-values lower than 0.0001 and 0.001). Interestingly, benznidazole treatment resulted in downregulation of these genes only in the CCC-Pos (with *p* values = 0.001 for *IL2RA* and 0.01 for *FCER*2, *IL10*, and *IL1B*), but not in IND-Pos patients, in which treatment-induced upregulation of these genes although it was only statistically significant for the *IL13*. Treatment also induced in IND-Pos versus IND-Pre patients a statistically significant increase in the expression of *LGALS*9 and *PDCD1LG2* (*p-*values = 0.05 and 0.01, respectively) and also versus HD (*p-*values = 0.001 and 0.05, respectively). In addition, the treatment also induced in CCC patients an upregulation of the *LGALS9* gene (*p-*value = 0.05). By contrast, treatment induced in CCC patients a downregulation of *PDCD1LG2* although the drop had no statistical significance. Together, these data suggest that treatment has an important effect on the immune system of the Chagas disease patients, although its influence depends on the status and severity of the disease of the patients.

## 4 Discussion

The quality and multifunctional capacity of the antigen-specific T-cell response are crucial in determining the disease outcome of *T. cruzi* infection. Several studies show that *T. cruzi*-infected mammalian cells produce cytokines and chemokines that act in the early stages of infection, promoting a defensive response against the parasite (Rezende-Oliveira et al., 2012). These molecules participate in the control of parasitism but also contribute to the establishment of chronic inflammatory lesions in various target tissues, most commonly leading to severe myocarditis. Understanding the specific immune response generated during the infection and how current antiparasitic drugs influence the parasitemia and the mechanisms that may or may not control parasite persistence and the pathogenesis of the illness will undoubtedly aid in preventing, modulating, and controlling the disease suffered by 7 million people worldwide.

For this purpose, and taking into account that the immune system plays an important role in therapeutic success, a high-throughput real-time qPCR analysis has been performed to simultaneously determine global changes in gene expression profiles of 106 immune system-related genes after benznidazole treatment in antigen-restimulated PBMC from chronic Chagas disease patients (IND and CCC I). The results shown here indicate that benznidazole treatment influences the expression pattern and level of several genes coding for cytokines, chemokines, their receptors, adhesion molecules, phenotype markers, transcription factors, cytotoxic molecules, molecules involved in apoptosis and senescence, dendritic cell markers, as well as inhibitory receptors and their ligands. This effect has particularities on the expression of some immunological molecules, depending on the phase and severity of the disease. Particularly in CCC I patients, a regulation of the expression level of some genes to a similar level to that of healthy subjects suggests a beneficial effect of treatment in CCC I patients. Thus, in this study, in treated patients, a variation in the expression level of seven (in IND) and 15 (in CCC I) out of the 106 genes analyzed was detected versus nontreated IND and CCC I patients, respectively.

The expression levels of *IDO1*, *IL13*, *PDCD1LG2*, and *TGFB2* increased in IND patients as a consequence of treatment. Genes such as those encoding enzymes such as IDO1, which was upregulated in IND versus HD and CCC I versus HD, continued increasing their expression after treatment in IND patients versus untreated. Modifications of the expression level of *IDO1* in treated CCC I patients were not detected. This is an interferon-induced tryptophan-catabolizing enzyme (Hassanain et al., 1993) with the ability to inhibit the proliferation of intracellular pathogens. Although little is known about the role of IDO1 in parasitic infections, it could play a regulatory role in preventing an exacerbated immune response. Thus, *IDO1* expression has been shown to be upregulated during *T. cruzi* infection in mice, resulting in its activity being critical to controlling this parasite replication during the acute phase of infection (Knubel et al., 2010).

The *PDCD1LG2* gene coding for programmed cell death 1 ligand 2 (PD-L2) was also upregulated after treatment in IND. PDL2 has been shown to have a protective role in *T. cruzi* infection by controlling the Arg I/iNOS balance in favor of iNOS being thus a key element in the control of *T. cruzi* replication in macrophages (Dulgerian et al., 2011). Thus, treatment of peritoneal macrophages from *T. cruzi*-infected mice with anti-PD-L2 blocking antibody showed a reduction in iNOS expression and in NO production and an increase in arginase expression and activity (Dulgerian et al., 2011). In addition, PD-L2 KO-infected mice showed higher parasitemia than WT-infected mice (Dulgerian et al., 2011). Upregulation of the *IL-13* coding gene in IND patients after treatment was also detected. Traditionally, IL-4 and IL-13 are associated with Th2-dependent susceptibility to intracellular infections. Consistent with this is the fact that transgenic mice overexpressing IL-13 are highly susceptible to *T. cruzi* infection with enhanced parasitemia and impaired survival compared to wild-type mice (Dar and Hölscher, 2018). However, the central dogma of Th1 and Th2 cells and their signature cytokines, INF gamma and IL-4/IL-13 related to resistance or susceptibility to infection is changing toward interconnected pathways in which depending on the disease model, the IL-4 and even the IL-13 can mediate Th1 immunity (Hurdayal and Brombacher, 2014). In addition, upregulation of the *TGFβ2* gene has been detected in treated IND patients. In spite of the important role of TGF-*β* in the establishment and development of chagasic cardiomyopathy, little is known about its expression and role in asymptomatic patients (Ferreira et al., 2022). Further research is needed to improve the knowledge of this transforming growth factor in Chagas disease.

Benznidazole influences the downregulation of many genes. *CCL5*, *GZMH*, and particularly *IL17A* showed a decrease in their expression level following the treatment of IND and CCC I patients. In CCC I patients, the effect of treatment was stronger than in IND in terms of downregulation of cytokine expression levels. In addition, in CCC I-treated patients, there was also a substantial downregulation of *CCR1*, *FCER2*, *GZMA*, *HAVCR2*, *IL10*, *IL1B*, *IL12RA*, *ITGAX*, and *PFR1* genes, and particularly the gene expression of *CCR1*, *GZMA*, *ITGAX*, and *PRF1* genes reached half the amount of expression detected in nontreated CCC I patients. Conversely, *LGALS9*, *STAT1*, and *TNFSF10* genes had increased their expression in CCC-Pos versus CCC-Pre patients. Interestingly, the expression level of some of these cytokines in treated IND and CCC I patients was consistent with the expression level seen in healthy donors. That is the case of *GZMH*, whose expression level was upregulated in IND and CCC I chronic patients versus HD and showed to be significantly downregulated in IND and CCC I patients after treatment. In addition to the drop in *GZMH* expression, a striking downregulation of *GZMA* and *PRF1* was also detected in CCC I patients after treatment. These data support that treatment has an important effect on parasite survival, the control or reduction of the parasite load, and consequently on the activation of the CD8^+^ T cells, reducing their cytotoxic capacity to release cytotoxic granules, composed of granzymes and perforins. This is consistent with the GSEA presented here, which revealed a positive correlation between CCC-Pre (and a negative correlation between CCC-Pos) and the immune pathway “CTL-mediated immune response against target cells (BIOCARTA_CTL_PATHWAY)”.

Treatment also induced downregulation of the expression of genes coding for various pro-inflammatory markers such as the CCL5 chemokine (also known as RANTES) and IL-1β cytokine in both IND and CCC I patients. Additionally, in CCC I, the treatment also downregulates the expression of the CCL5/RANTES receptor, CCR1. *CCL5* is primarily expressed by T cells, as well as monocytes, neutrophils, platelets, macrophages, endothelial, and epithelial cells (Appay and Rowland-Jones, 2001). This chemokine induces the recruitment of T cells, dendritic cells, monocytes, NK cells, and other cell types to the site of inflammation and infection by binding to specific receptors, mainly CCR5 (Levy, 2009). CCL5 has been shown to play a key role in the immune response to viral infection, as it is degranulated from activated HIV-specific CD8^+^ T cells along with perforin and granzyme A (Wagner et al., 1998). In addition, CCL5 has been proposed to be important as a general B-cell coactivator, making it a potential target for novel treatment strategies for autoimmune and other inflammatory diseases. Previous studies have shown that chronic infection in Chagas disease patients promotes a progressive and important upregulation of *CCR5* expression in IND and, particularly, in CCC I patients, which led to proposing it as a marker of disease progression and severe pathology (Gómez et al., 2023). Herewith, as a result of benznidazole treatment, a downregulation of *CCL5* is observed in both IND and CCC I patients, supporting a decreased cytotoxic activity of CD8^+^ T cells in treated patients and a reduction of inflammation, which is directly related to a decrease in infection-induced cardiac tissue damage. This could be related to the effective and beneficial effect of the treatment with benznidazole in CCC I.

The major drop following treatment in both IND and CCC I patients *versus* untreated patients has been related to the expression level of *IL17A*, mostly known as IL17. This pro-inflammatory cytokine is produced by various cell types, including neutrophils, dendritic cells, B cells, and T cells, in addition to CD4 Th17 cells, varying its immune role between different organs. The current understanding of the role of Th17 cells in the progression of pathogenesis or their contribution to host-protective immunity is rapidly evolving. Recent evidence indicates that IL-17 has crucial tissue-dependent roles in maintaining health during response to injury, physiological stress, and infection (McGeachy et al., 2019). By contrast, IL-17 has also been considered a pathogenic cytokine in several inflammatory diseases, autoimmune diseases, and viral and parasite infections (Tesmer et al., 2008). Using mice deficient in IL-17RA, it has been shown that this cytokine is required for host protection against the infection caused by *T. cruzi* in the acute phase of the disease (Boari et al., 2012). IL-17RA is necessary for the recruitment of neutrophils that destroy the parasite and also regulate inflammatory responses and collateral tissue damage by secreting IL-10 (Boari et al., 2012). In addition, data raise the possibility that IL-17A plays a crucial immunomodulatory role in the chronic phase of Chagas disease and might be involved in protection against myocardial damage (Magalhães et al., 2013).

IL-17A level has been proposed as a marker for benznidazole efficacy since, before treatment, seropositive children have shown higher plasma levels of IL-17A than seronegative children. Twelve months after treatment, these high levels decreased to seronegative levels in children with recurrent negative conventional and quantitative real-time PCR and did not decrease in those children with a positive PCR result, who showed significantly higher levels of IL-17A after treatment (Velásquez et al., 2019). There are still many open questions regarding the role of IL-17A in *T. cruzi* infection, and further studies are needed concerning the Th17 response in naturally infected humans with *T. cruzi*. The data shown here, involving a significant downregulation of IL-17 in treated patients, support a beneficial effect of benznidazole in both IND and CCC I patients.

The expression of proinflammatory IL-1β also decreases after treatment in CCC I patients. IL-1β is rapidly induced in response to *T. cruzi* and promotes inflammatory activity in acute experimental myocarditis induced by the parasite (Chandrasekar et al., 1998). It has also been reported that IL-1β is a critical soluble mediator of the overall hypertrophic response for both parasite-infected and uninfected cell populations (Petersen and Burleigh, 2003). Other authors using an experimental murine model of *T. cruzi* infection with *Il-1r^−/−^* and wild-type mice have suggested that IL-1β is not critical to the generation/maintenance of cardiac arrhythmias or systolic disfunction found in CCC patients (Sousa Oliveira et al., 2022).

The observed downregulation in the expression level of *IL1β* in CCC I-treated patients, together with the decreased expression of *CCL5*, *IL10*, and *IL2RA*, has been related to the IL18 pathway, as deduced from STRING analyses. Since IL-18 induces cardiomyocyte hypertrophy (Chandrasekar et al., 2005) and increased fibronectin expression (Reddy et al., 2008), high levels of IL-18 are expected to lead to cardiac hypertrophia and fibrosis, with a deleterious impact on heart function. A potential benefit of targeting IL-18 as a therapy in Chagas cardiomyopathy has been proposed based on the observed association between the *IL18*-607 AA genotype (to which low IL-18 production is associated) and decreased risk of developing cardiomyopathy in Chagas disease patients (Gomes dos Santos et al., 2020). A protective effect of the *IL1B*-31 TC genotype against cardiac disease severity has also been reported (Gomes dos Santos et al., 2020). The effect of treatment in CCC I patients also leads to downregulation of the cytokines involved in IL-4 and IL-13 signaling (*IL1B*, *IL10*, *IL17A*, FCER2, and *ITGAX*) and IL-10 signaling (*IL1B*, *CCL5*, *IL10*, *FCER2*, *CCR1*) as deduced from the protein–protein interaction network constructed using the STRING platform.

*IL2RA* expression levels also deceases in CCC I patients after treatment. The role of IL-2 during *T. cruzi* infection is not established. IL-2 may be a key cytokine involved in promoting or downregulating immune responses, probably in a dose-dependent manner. IL-2 activates NK cells, CD4, and CD8 T cells and is required for the expansion and maintenance of regulatory T cells. Blocking of IL-2 during the acute *T. cruzi* infection by using a neutralizing monoclonal antibody resulted in lower parasitemia and mortality of treated animals (Nihei et al., 2021). The benznidazole treatment has influenced an additional marked downregulation of the TIM-3 encoding gene, *HAVCR2*, in IND and particularly in CCC I patients. The soluble form of immune checkpoint TIM-3 (sTIM-3) has been identified in the blood of patients with a large diversity of pathologies, including various cancers, infectious diseases, and chronic inflammatory diseases, and its expression has been associated with a worse prognosis, which could be used as diagnostic and therapeutic marker (Bailly et al., 2023). TIM-3 can positively or negatively regulate innate and adaptive immune responses, depending on the cell type and immunological scenario in which it is engaged. The expression of TIM-3 was found to be increased *ex vivo* in CD8^+^ (Lasso et al., 2015; Pérez-Antón et al., 2020) and CD4^+^ (Pérez-Antón et al., 2021) T cells from patients with Chagas disease *versus* HD, predominantly in patients with cardiac symptoms. This dysfunctional process and the exhaustion of T cells during chronic Chagas disease have been shown to be reversed, at least partially after treatment of patients with benznidazole (Mateus et al., 2017; Pérez-Antón et al., 2021), since expression and coexpression by CD8^+^ T cells of inhibitory receptors TIM-3, 2B4, CD160, PD-1, and CTLA-4 was reduced in asymptomatic and cardiac Chagas disease patients (Pérez-Antón et al., 2020). Consistent with these data are those presented here regarding a reduction of *TIM3* expression in treated CCC I patients. By contrast, the Gal-9 coding gene (*LGALS9*), one of the four TIM3 ligands, was found to be upregulated in CCC I-treated patients versus nontreated CCC I patients, together with the *STAT1* and *TNFSF10* genes. The *LGALS9* expression level was not detected in either IND or HD subjects. T-cell receptors and their ligands, such as the TIM3/Gal-9 system, are key players in upholding the balance between pro- and anti-inflammatory signals and play a major role in regulating adaptative immunity against different pathogens (Bailly, 2023). In spite of the fact that Gal-9 is recognized as a valuable biomarker for assessing the severity of various infectious diseases, its specific function and significance in Chagas disease remain to be determined (Meira et al., 2023).

STAT1, a proinflammatory transcription factor, plays a key role as an immune response modulating factor by lessening both the early, inflammatory responses of innate immunity and the sustained, destructive actions of adaptive immunity (Jung et al., 2020). STAT1 has been shown to have a critical role in controlling *T. cruzi* infection since STAT1 knockout mice are more susceptible to *T. cruzi* infection than wild-type animals, presenting higher parasitemia in blood and tissues and higher mortality (Kulkarni et al., 2015). Herewith, an upregulation of the *STAT1* gene has been detected in treated versus nontreated CCC I patients, which may be considered a beneficial effect of the benznidazole in CCC I patients. However, its precise role in *T. cruzi* infection control has not been previously reported, although IL-17, which undergoes STAT-1-dependent regulation (Villarino et al., 2010), has been shown to be important for controlling the inflammation and mortality associated with *T. cruzi* infection (Bermejo et al., 2013). This is consistent with the data showing that splenic antigen-driven IL-17 was upregulated in infected STAT-1 KO mice, which had approximately twofold more resident neutrophils than WT mice (Kulkarni et al., 2015).

*TNFSF10* has shown an important upregulation in CCC I after treatment. This molecule, also known as TNF-related apoptosis-inducing ligand (TRAIL), was originally identified by its high sequence homology to FasLigand/CD95L and TNF-α. TNF-α, TRAIL, and FASLigand/CD95L have been implicated in the cell death pathways leading to distinct levels of heart dysfunction. TNF-α and TRAIL have been proposed as potential predictor markers of severe ventricular systolic function in CCC since high plasma concentrations of TNF-α and TRAIL have been strongly correlated with severe CCC (Lula et al., 2009). Interestingly, the interferon cytokine family has been shown to have an important role in the regulation of TRAIL expression via phosphorylation of STAT-1 (Miura et al., 2006), which is one of the three genes mainly upregulated in CCC I patients after treatment.

The data shown here support that in IND and CCC I patients, treatment with benznidazole downregulates the proinflammatory and cytotoxic responses triggered as a consequence of *T. cruzi* infection, and probably prevents marked tissue damage and healing, evidencing its positive effect on the control of the pathology of IND and CCC I patients. Moreover, this study revealed that most of the genes downregulated in cardiac patients after treatment had been shown to be upregulated in untreated CCC I patients versus HD (Gómez et al., 2023). The modulated pathways in which the upregulated genes participated in CCC I patients versus HD were the same three immunological pathways (IL-10 signaling [FDR = 5.83*e*−17]; IL-4 and IL-13 signaling [2.75*e*−13]; and IL-18 signaling [FDR = 8.75*e*−13]) than those downregulated in treated versus untreated CCC I patients (**Supplementary Figure S2**). This was taken as an indication of a beneficial effect of benznidazole as it induced homeostasis of the immune system probably by an effect on the parasitemia rate. Additional analyses employing a broader battery of molecules involved in the antigen-specific immune response and signaling pathways will highlight mechanisms related to the establishment and also to the control of *T. cruzi* infection following treatment. Furthermore, their knowledge will be of enormous value for vaccine development, drug design, and as biomarkers of disease progression and treatment efficacy.

## Data availability statement

The datasets presented in this study can be found in online repositories. The acess link is https://saco.csic.es/s/TWSx6fNzo8xnLBz.

## Ethics statement

The protocols employed in this study were approved by the Ethics Committees of the Consejo Superior de Investigaciones Científicas (Spain - Reference: 013/2020) and of the Hospital Virgen de la Arrixaca (Murcia, Spain - Reference: 2020-1-11-HCUVA). An informed consent was signed by all voluntary patients prior to their inclusion in the study. The studies were conducted in accordance with the local legislation and institutional requirements. The participants provided their written informed consent to participate in this study.

## Author contributions

IG: Formal analysis, Methodology, Writing – original draft, Writing – review & editing. AE: Formal analysis, Methodology, Writing – review & editing. GP: Formal analysis, Methodology, Writing – review & editing. BC: Methodology, Writing – review & editing. CB: Methodology, Writing – review & editing. MSi: Methodology, Writing – review & editing. MSe: Funding acquisition, Writing – review & editing. EC: Formal analysis, Funding acquisition, Methodology, Writing – review & editing. ML: Conceptualization, Formal analysis, Funding acquisition, Methodology, Project administration, Validation, Writing – original draft, Writing – review & editing. MT: Conceptualization, Formal analysis, Funding acquisition, Methodology, Project administration, Validation, Writing – original draft, Writing – review & editing.
