# Supplementary material for: The expression of immune response genes in patients with chronic Chagas disease is shifted toward the levels observed in healthy subjects as a result of treatment with Benznidazole
Source: Front Cell Infect Microbiol. 2024 Jul 23;14:1439714. doi: 10.3389/fcimb.2024.1439714 (PMC11307780; doi:10.3389/fcimb.2024.1439714)
Supplement: Supplementary file 1 [file Supplementaryfile_1.docx]

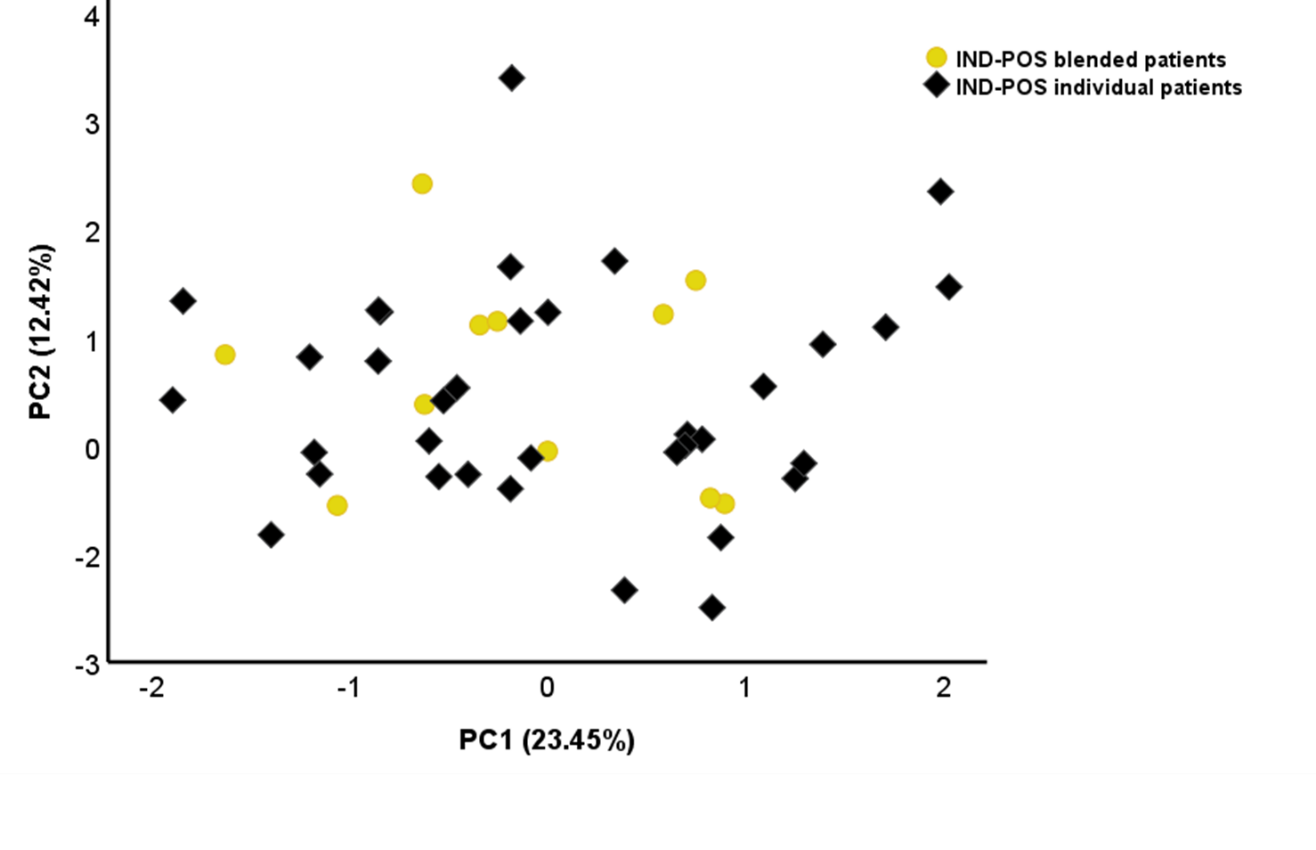


**B**

**A**


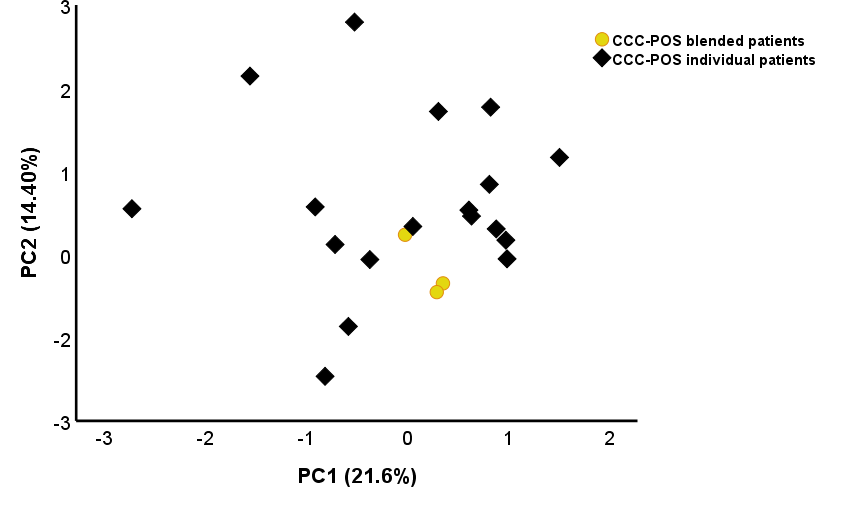


**Supplementary Figure 1**. Principal-component analysis of NRQ (Normalized Relative Quantities) values of gene expression of 106 analyzed genes in (**A**) post-treatment IND blended patients (yellow circles) and IND individual patients (black rhombus) and in (**B**) post-treatment CCC patients (yellow circles) and CCC individual patients (black rhombus). PC1 and PC2 are plotted on the x and y axes, respectively, and the proportion of variance captured for both components is given as a percentage. These results shown were confirmed by a two-tailed unpaired t-test showing that there were no statistically significant differences between the scores obtained in the two groups for each represented principal component in both groups of patient (PC1 and PC2 *p* = 0.61 for A and PC1 *p* = 0.71, PC2 *p* = 0.21 for B).
