# Supplementary material for: The expression of immune response genes in patients with chronic Chagas disease is shifted toward the levels observed in healthy subjects as a result of treatment with Benznidazole
Source: Front Cell Infect Microbiol. 2024 Jul 23;14:1439714. doi: 10.3389/fcimb.2024.1439714 (PMC11307780; doi:10.3389/fcimb.2024.1439714)
Supplement: Supplementary file 2 [file Supplementaryfile_2.docx]

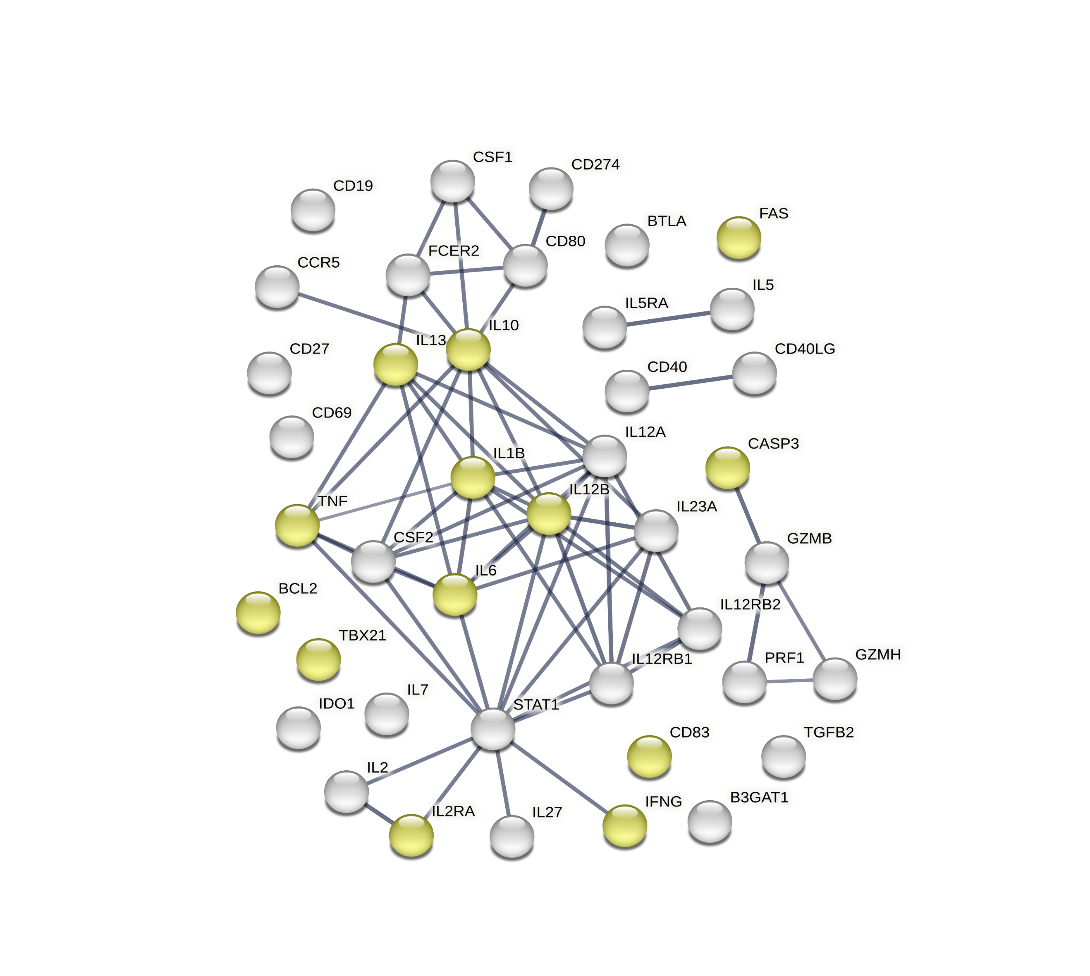

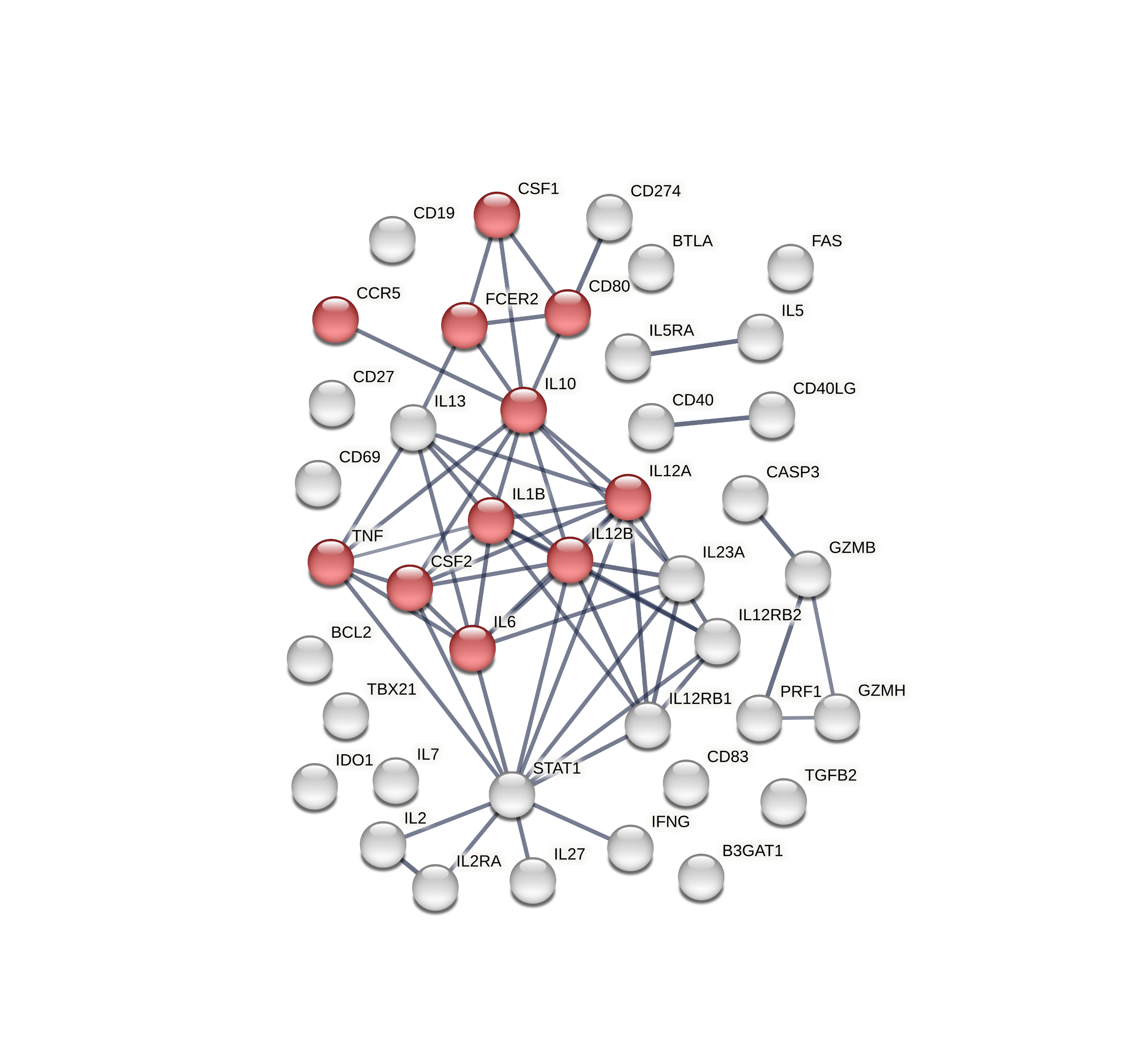

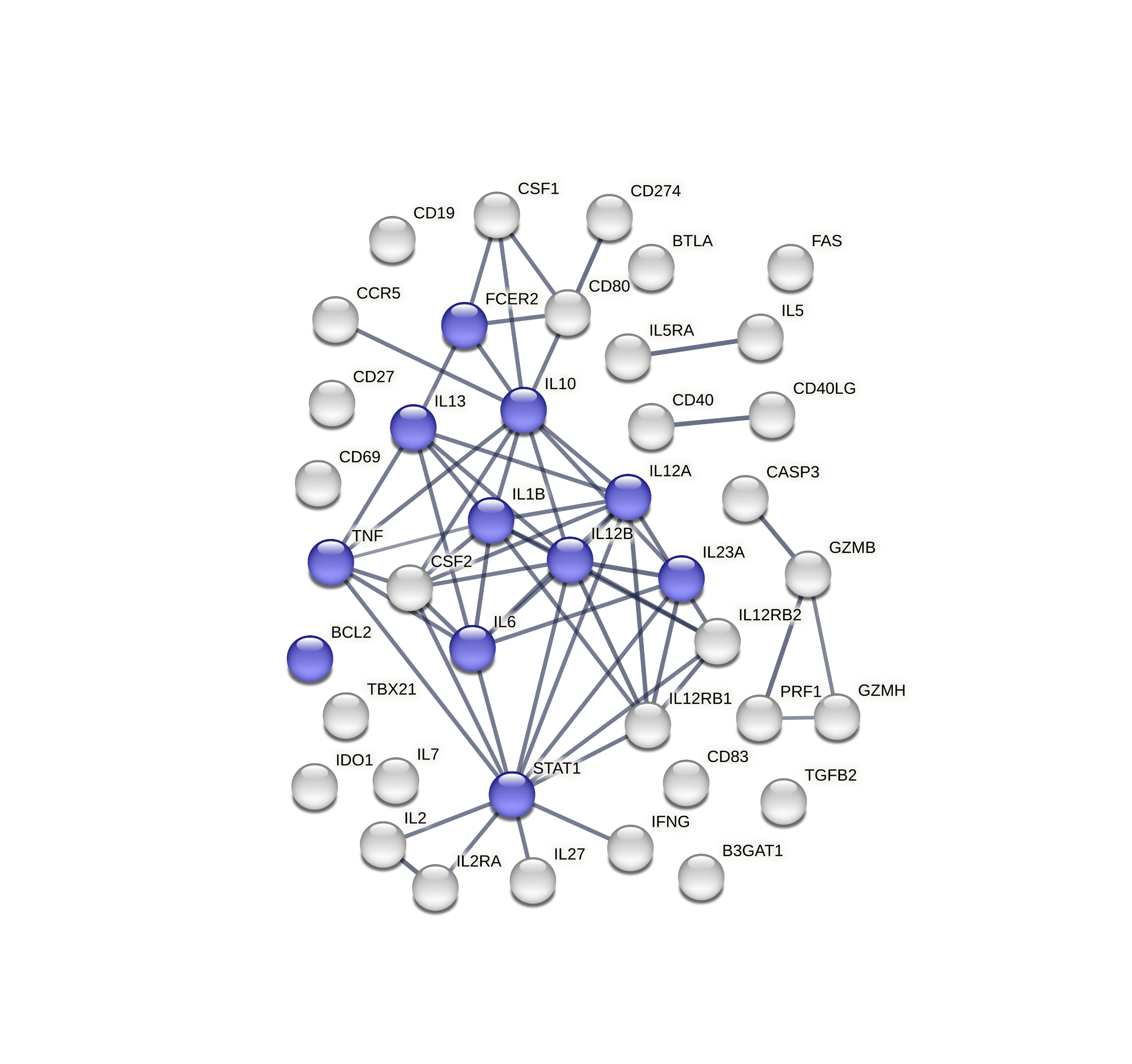


**A**

**B**

**C**

IL-10 signaling

IL-4 and IL-13 signaling

IL-18 signaling pathway

**Supplementary Figure 2. STRING Protein-protein interaction analysis representing the proteins encoded by downregulated genes in CCC *versus* HD**. The linkage of each node represents the interactions (edges) reported for these molecules in different immunological processes/pathways; PPI enrichment *p*-value < 1.0e-16. Network edges represent confidence (line thickness indicates the strength of data support). PPI network was constructed setting the confidence score threshold at the high level (0.7) and active interaction sources, including data from published experiments, databases, co-occurrence, gene fusion, neighborhood and co-expression, species limited to “*Homo sapiens*”. Proteins are described to be involved in specific pathways: IL-10 signaling (HSA-6783783; FDR = 5.83e-17) (red), IL-4 and IL-13 signaling (HSA-6785807; FDR = 2.75e-13) (blue) and IL-18 signaling pathway (WP4754; FDR = 8.7e-13) (yellow).
