# Supplementary material for: The expression of immune response genes in patients with chronic Chagas disease is shifted toward the levels observed in healthy subjects as a result of treatment with Benznidazole
Source: Front Cell Infect Microbiol. 2024 Jul 23;14:1439714. doi: 10.3389/fcimb.2024.1439714 (PMC11307780; doi:10.3389/fcimb.2024.1439714)
Supplement: Supplementary file 3 [file Table_1.docx]

**Supplementary table 1. PC1-correlated genes**. Genes with factor loading of Principal Component 1 (PC1) higher than 0.6 or lower than -0.6 from the Principal Component Analysis (PCA) applied on the normalized relative quantities (NRQ) of indeterminate Chagas disease patients pre- and post-treatment.

| **Gene** | **Factor loading for PC1** |
| --- | --- |
| *CD2* | 0.893 |
| *ITGA4* | 0.866 |
| *GATA3* | 0.844 |
| *ITGAL* | 0.843 |
| *TGFBR2* | 0.819 |
| *IL2RG* | 0.809 |
| *ICOS* | 0.802 |
| *CD28* | 0.769 |
| *IL18R1* | 0.728 |
| *CD40LG* | 0.719 |
| *CD4* | 0.715 |
| *CD8A* | 0.693 |
| *IL12RB1* | 0.684 |
| *CD69* | 0.682 |
| *CTLA4* | 0.681 |
| *CD3E* | 0.669 |
| *GZMK* | 0.638 |
| *KLRG1* | 0.622 |
| *CD274* | -0.629 |
| *IFNGR2* | -0.640 |
| *IDO1* | -0.646 |
| *PDCD1LG2* | -0.684 |
| *ICAM1* | -0.705 |
| *ITGAX* | -0.708 |
