# Supplementary material for: The expression of immune response genes in patients with chronic Chagas disease is shifted toward the levels observed in healthy subjects as a result of treatment with Benznidazole
Source: Front Cell Infect Microbiol. 2024 Jul 23;14:1439714. doi: 10.3389/fcimb.2024.1439714 (PMC11307780; doi:10.3389/fcimb.2024.1439714)
Supplement: Supplementary file 4 [file Table_2.docx]

**Supplementary table 2. PC2-correlated genes**. Genes with factor loading of Principal Component 1 (PC1) higher than 0.6 or lower than -0.6 from the Principal Component Analysis (PCA) applied on the normalized relative quantities (NRQ) of cardiac Chagas disease patients pre- and post-treatment.

| **Gene** | **Factor loading for PC2** |
| --- | --- |
| *FASLG* | 0.879 |
| *CCL5* | 0.787 |
| *B3GAT1* | 0.747 |
| *HAVCR2* | 0.741 |
| *PRF1* | 0.729 |
| *GZMH* | 0.708 |
| *GZMA* | 0.680 |
| *TGFBR1* | 0.642 |
| *IL10RA* | 0.633 |
| *NCAM1* | 0.625 |
| *GZMB* | 0.625 |
| *LAG3* | 0.623 |
| *STAT1* | -0.680 |
