# Supplementary material for: The expression of immune response genes in patients with chronic Chagas disease is shifted toward the levels observed in healthy subjects as a result of treatment with Benznidazole
Source: Front Cell Infect Microbiol. 2024 Jul 23;14:1439714. doi: 10.3389/fcimb.2024.1439714 (PMC11307780; doi:10.3389/fcimb.2024.1439714)
Supplement: Supplementary file 5 [file Table_3.docx]

**Supplementary Table 3.** Expression level of differentially expressed genes in IND and/or CCC I Chagas disease patients pre and post-treatment. Average expression values expressed as NRQ (Normalized relative quantities) identified in healthy donors (HD), indeterminate patients Pre-treatment (IND-Pre) and Post-treatment (IND-Pos) and cardiac patients Pre-treatment (CCC-Pre) and Post-treatment (CCC-Pos). In bold are highlighted the genes whose post-treatment expression has a value that tends to that of healthy donors and/or, in the case of CCC, to values of IND patients.

| Gene | HD | IND-Pre | IND-Pos | CCC-Pre | CCC-Pos |
| --- | --- | --- | --- | --- | --- |
| ***CCL5*** | **1.188** | **1.485** | **0.898** | **1.298** | **0.844** |
| *CCR1* | 3.339 | 1.166 | 1.102 | 1.156 | 0.447 |
| ***FCER2*** | **0.684** | **1.214** | **1.323** | **1.754** | **1.012** |
| *GZMA* | 2.139 | 1.262 | 0.865 | 1.524 | 0.701 |
| ***GZMH*** | **0.828** | **1.577** | **0.934** | **1.641** | **0.947** |
| *HAVCR2* | 2.351 | 0.939 | 0.970 | 1.149 | 0.703 |
| *IDO1* | 0.408 | 1.137 | 1.775 | 1.305 | 1.588 |
| ***IL10*** | **0.843** | **1.050** | **1.207** | **1.605** | **0.894** |
| ***IL13*** | **0.694** | **0.992** | **1.593** | **1.399** | **0.908** |
| *IL17A* | 0.604 | 0.503 | 0.159 | 0.462 | 0.000 |
| ***IL1B*** | **0.671** | **1.249** | **1.517** | **1.903** | **1.103** |
| ***IL2RA*** | **0.634** | **1.109** | **1.350** | **1.439** | **0.819** |
| *ITGAX* | 3.214 | 0.966 | 1.022 | 0.947 | 0.440 |
| ***LGALS9*** | **0.924** | **1.004** | **1.465** | **0.706** | **1.109** |
| ***PDCD1LG2*** | **1.033** | **0.921** | **1.424** | **1.232** | **0.889** |
| *PRF1* | 0.928 | 1.121 | 1.056 | 1.534 | 0.619 |
| *STAT1* | 0.499 | 1.353 | 1.451 | 1.144 | 1.749 |
| *TGFB2* | 0.425 | 0.625 | 1.208 | 0.751 | 1.118 |
| *TNFSF10* | 0.592 | 1.075 | 1.151 | 0.825 | 1.368 |
